# A Putative Lipoprotein Mediates Cell-Cell Contact for Type VI Secretion System-Dependent Killing of Specific Competitors

**DOI:** 10.1128/mbio.03085-21

**Published:** 2022-04-11

**Authors:** Lauren Speare, Madison Woo, Anne K. Dunn, Alecia N. Septer

**Affiliations:** a Department of Earth, Marine and Environmental Sciences, University of North Carolina at Chapel Hill, Chapel Hill, North Carolina, USA; b Department of Microbiology, Oregon State University, Corvallis, Oregon, USA; c Department of Microbiology and Plant Biology, University of Oklahoma, Norman, Oklahoma, USA; Max Planck Institute for Marine Microbiology

**Keywords:** symbiosis, type VI secretion, *Aliivibrio fischeri*, lipoprotein, symbiosis

## Abstract

Interbacterial competition is prevalent in host-associated microbiota, where it can shape community structure and function, impacting host health in both positive and negative ways. However, the factors that permit bacteria to discriminate among their various neighbors for targeted elimination of competitors remain elusive. We identified a putative lipoprotein (TasL) in *Vibrio* species that mediates cell-cell attachment with a subset of target strains, allowing inhibitors to target specific competitors for elimination. Here, we describe this putative lipoprotein, which is associated with the broadly distributed type VI secretion system (T6SS), by studying symbiotic Vibrio fischeri, which uses the T6SS to compete for colonization sites in their squid host. We demonstrate that TasL allows V. fischeri cells to restrict T6SS-dependent killing to certain genotypes by selectively integrating competitor cells into aggregates while excluding other cell types. TasL is also required for T6SS-dependent competition within juvenile squid, indicating that the adhesion factor is active in the host. Because TasL homologs are found in other host-associated bacterial species, this newly described cell-cell attachment mechanism has the potential to impact microbiome structure within diverse hosts.

## INTRODUCTION

Microbial communities perform important ecosystem functions that impact host health and drive essential biogeochemical processes on our planet. However, these microbial communities do not assemble as peaceful, coexisting populations. Indeed, recent studies suggest that competition among microbes is more prevalent than cooperation (1, 2), and many microbial populations actively interfere with their competitors’ abilities to access limited resources or colonize an ecological niche. A variety of interference competition strategies have been described, including contact-dependent and diffusible mechanisms (3, 4). Contact-dependent mechanisms require an inhibitor cell to physically interact with a competitor cell to deliver toxic effectors (5–7), and such strategies may be particularly useful in liquid environments, where diffusible antimicrobial molecules can quickly become diluted.

For some contact-dependent systems, the molecules that mediate cell-cell contact also allow inhibitor cells to discriminate between different cell types, so that only cells with the appropriate identifying surface molecule(s) are targeted for elimination (8–12). Because these receptor-ligand interactions, which occur across cell surfaces, promote killing of only certain cell types, the competitive outcomes of these interactions can substantially alter diversity within a microbial community. Although the underlying mechanisms of targeted killing have been elucidated for a few systems (10, 13, 14), most remain unknown. One example of the latter is the broadly distributed type VI secretion system (T6SS). Several studies of this contact-dependent killing system have reported that not all bacterial populations are susceptible to its lethal capabilities (15–17), suggesting that T6SS-encoding cells may discriminate among potential competitor cells to target specific bacterial populations for elimination.

**FIG 1:**
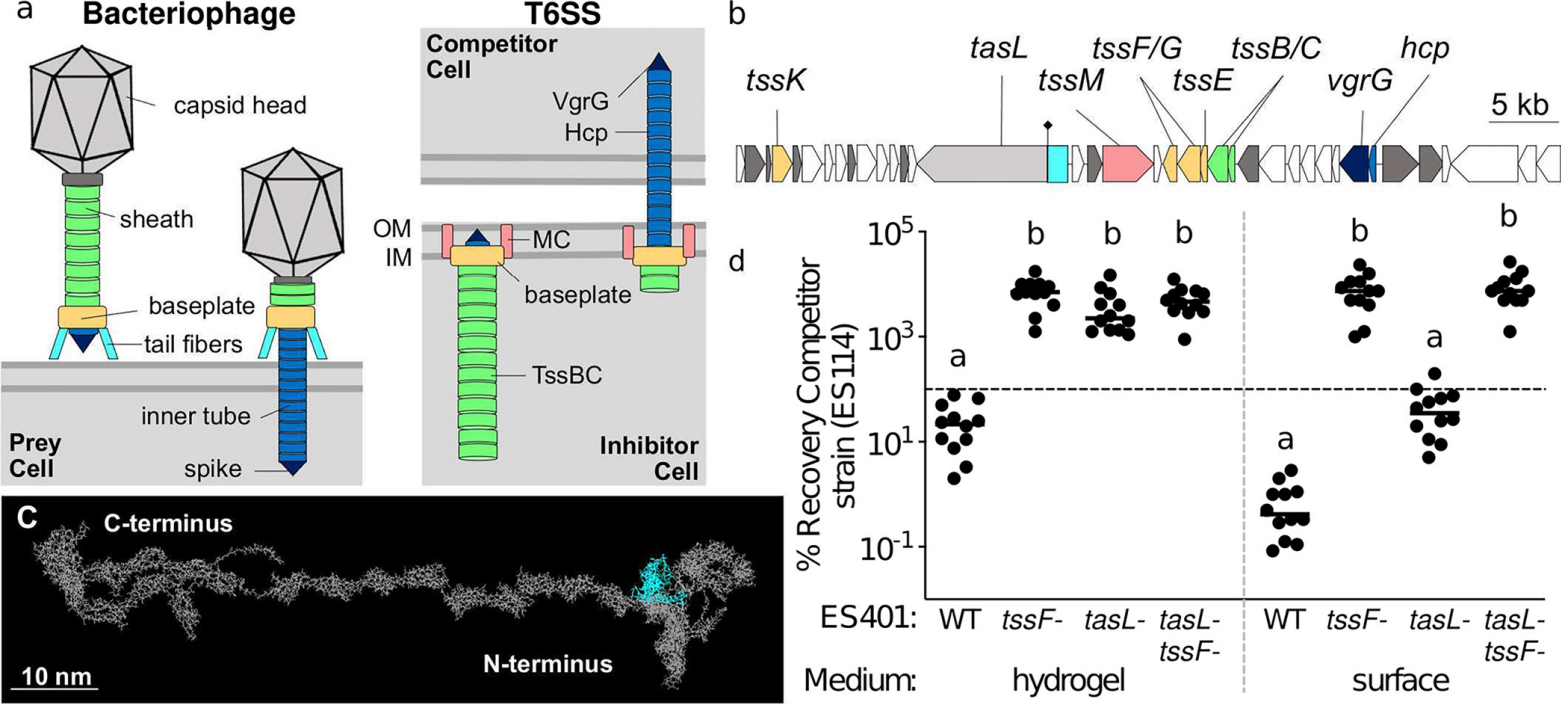
TasL is necessary for killing in hydrogel. (a) Schematic models of extended (left) and contracted (right) bacteriophage and T6SS. Homologous components are shown in the same color. MC, membrane complex; OM, outer membrane; IM, inner membrane. (b) Gene map of the V. fischeri T6SS2 genomic island from ES401. Gray and colored genes are conserved T6SS genes, and genes of unknown function are in white. The putative lipoprotein gene *tasL* (VFES401_15750) is in gray and cyan. The black diamond at ∼1.3 kbp indicates the location of a disruption mutation. (c) Predicted structure of TasL using RaptorX. The entire structure is shown in gray, and the structure remaining within the *tasL* disruption mutation is shown in cyan. (d) Percent recovery of the competitor strain (ES114) from coincubation assays with the ES401 wild-type (WT), *tssF* mutant, *tasL* mutant, or *tasL tssF* double mutant strains incubated in hydrogel or on agar surfaces for 12 h. The horizontal line indicates 100% recovery. Letters indicate significantly different percent recovery of ES114 incubated with different ES401 strains under the same conditions (two-way analysis of variance [ANOVA] with Dunnett’s multiple-comparison posttest; *P* < 0.0001). Each experiment was performed three times on separate days and with separate cultures, and combined data are shown (*n* = 12).

T6SS genes are prevalent in genomes of Gram-negative bacteria (18–20) and have been functionally characterized in diverse species, including environmental, pathogenic, and symbiotic bacteria (17, 18, 21–32). The T6SS resembles an inverted phage (Fig. 1a) that functions like a molecular syringe to deliver effector proteins directly into target cells (33). Recent work suggests that T6SS activity impacts the composition and spatial distribution of natural microbial communities living within diverse hosts, from plants to marine invertebrates and humans, underscoring the importance of T6SS-mediated competition as a driver of microbiome assembly and function (19, 21–23, 34–39). T6SSs use effector proteins that have diverse functional activities, including the ability to break down cell membranes, cell walls, and DNA (1, 40, 41). Because T6SS effectors often degrade conserved cellular structures, it is predicted that specificity of T6SS killing may come into play at the cell contact and/or effector delivery step (15, 42, 43), though other defense mechanisms have been described (44).

Although the molecular interactions underlying the observed specificity of T6SS-mediated killing are unclear, clues to this important knowledge gap may lie in the evolutionary history of this killing system. T6SSs are thought to have evolved from bacteriophages (45, 46) and therefore have many functional similarities to their phage relatives (Fig. 1a). Similar to bacteriophage, T6SSs use a molecular syringe to inject effectors into competitor cells (33). To ensure that the force needed to puncture the target cell for effector translocation is not transferred to pushing the cells away from one another, inhibitor cells must be able to sufficiently bind a competitor cell (33). Although such mechanisms have not yet been described for T6SSs, bacteriophage overcome this challenge with receptor-binding proteins (RBPs) that interact with specific receptors located on the bacterial surface (47, 48). RBPs, as well as tail fibers (47), can confer either a narrow or a broad host range, based on the allele of host receptor(s) as well as the density and localization of these receptors on the cell surface (49). Interestingly, a study using cryotomography to visualize the T6SS in Myxococcus xanthus identified large extracellular “antennae” clustered around the T6SS tip (50). Although the function of these structures is not yet known, the authors suggest that one possible function of these antennae, which are similar in appearance to bacteriophage tail fibers, could be to facilitate target cell recognition (50). Given these findings, and the shared evolutionary history of T6SSs and phage (46), it has been hypothesized that T6SS-encoding inhibitor cells may employ mechanisms similar to the phage RBP to establish contact with specific prey cells (33). Indeed, a recent study successfully engineered a receptor-ligand interaction to facilitate cell-cell contact for T6SS-mediated killing in liquid (51), demonstrating that such a hypothesis is theoretically sound. However, in order to directly test how coevolved competitors make contact in a liquid environment, a tractable model system is needed where competing bacterial populations deploy the T6SS under conditions that require biologically mediated cell-cell contact that can be studied both in culture and in the natural habitat.

Here, we used the symbiotic association between the bioluminescent bacterium Vibrio fischeri and Euprymna scolopes squid to explore T6SS-mediated competition in a natural system. V. fischeri uses several competitive mechanisms to compete for limited space within the host during colonization (52–54), including a strain-specific T6SS located on a genomic island on chromosome II (T6SS2) (Fig. 1b) (21). T6SS2 prevents incompatible strains from coexisting within the same crypt space, thereby shaping the diversity and spatial structure of the microbial community within a natural host, and this T6SS-mediated competition can be visualized and quantified in culture and in the host (21). Recently, we developed a high-viscosity liquid medium (hydrogel) that mimics host-like conditions and promotes formation of multistrain aggregates, which facilitates the cell-cell contact required for T6SS2-mediated killing (55). This new medium allowed us to examine how cells respond to simulated habitat transition from a lower-viscosity (aquatic) to higher-viscosity (host-like mucus) environment. When V. fischeri cells are transitioned from low- to high-viscosity medium, T6SS2 is highly expressed, multistrain aggregates form to facilitate inhibitor-competitor contact, and the T6SS is used to outcompete a target strain. However, the mechanism by which inhibitor cells facilitate contact with competitor cells for T6SS-mediated elimination was unknown. Here, we combined *in vitro* competition assays using our hydrogel culture medium with *in vivo* competitive colonization assays to determine how V. fischeri facilitates the cell-cell contact required for T6SS-mediated elimination of competitors in the host niche.

## RESULTS

### A large predicted lipoprotein is coordinately expressed with T6SS proteins.

To gain insight into how V. fischeri cells mediate contact for T6SS competition in hydrogel, we searched the proteomes of V. fischeri strain ES401 for proteins that were more abundant under conditions that promote cell-cell contact through aggregation (hydrogel) than conditions where V. fischeri does not form aggregates (liquid) (55). We identified a large (>380-kDa) putative lipoprotein (VFES401_15750; Fig. 1b) encoded in the T6SS2 genomic island that was highly expressed in hydrogel (0.00363 normalized spectral abundance factor percent [NSAF%]) relative to liquid (0.000750 NSAF%), which was similar to other T6SS2 proteins, such as the baseplate component TssF/VasA (shown in yellow in Fig. 1a) (0.00886 NSAF% in hydrogel and 0.00203 NSAF% in liquid) (55). The protein encoded in VFES401_15750 is predicted to localize to the outer membrane, based on the absence of a localization of lipoproteins (LOL) avoidance signal, which maintains inner membrane retention of lipoproteins (56). Because homologs of this putative lipoprotein were also found encoded in T6SS-containing gene clusters in other *Vibrio* and *Moritella* species (Table S1), we propose naming the VFES401_15750 gene product TasL, for “type VI secretion associated lipoprotein.” Furthermore, large predicted lipoproteins are also present in T6SS gene clusters of more diverse bacteria (Xanthomonas citri, Dyella thiooxydans, and Myxococcus xanthus) (Table S2). A SMART analysis of the TasL sequence identified regions near the C terminus of the protein that contained five repeat sequences with similarity to the plasma fibronectin type III domain, suggesting a potential function in cell adhesion and/or ligand binding (57). These protein sequence analyses, combined with a structural prediction of TasL using RaptorX (58), suggest that the N terminus of the protein may be anchored in the outer membrane while the adhesin domains in the C terminus may be located on the outside of the cell, where they could extend up to 70 nm from the cell surface (Fig. 1c). Therefore, we hypothesized that TasL may be important for promoting the cell-cell contact required for T6SS2-mediated killing.

10.1128/mBio.03085-21.6 TABLE S1 Distribution of V. fischeri TasL proteins associated with bacterial species associated with a marine host. Percent identity based on BLASTp results using VFES401_15750 (TasL) as the sequence query. Download Table S1, PDF file, 0.06 MB. Copyright © 2022 Speare et al. 2022 Speare et al. https://creativecommons.org/licenses/by/4.0/ This content is distributed under the terms of the Creative Commons Attribution 4.0 International license.

10.1128/mBio.03085-21.7 TABLE S2 Abundance of large lipoproteins in close proximity to T6SS gene clusters. Download Table S2, PDF file, 0.10 MB. Copyright © 2022 Speare et al. 2022 Speare et al. https://creativecommons.org/licenses/by/4.0/ This content is distributed under the terms of the Creative Commons Attribution 4.0 International license.

### TasL is required for T6SS-mediated competition in hydrogel.

To begin testing this hypothesis, we first used coincubation assays to determine the role of TasL in promoting T6SS2-dependent killing. We selected V. fischeri ES114 as the competitor strain because it does not encode the T6SS2 genomic island (21) and V. fischeri ES401 as the inhibitor strain because it kills ES114 using T6SS2 in coincubation assays on surfaces and in hydrogel (55). We chose to test the role of TasL using two different assays: (i) coincubations in a hydrogel medium, where cells must aggregate to facilitate the contact required for T6SS2-mediated killing, and (ii) coincubations on agar surfaces, where cells are forced into physical contact and aggregation factors are presumably not required. To make a *tasL* mutant, we introduced a disruption mutation in the beginning of the gene that would result in a truncated TasL protein composed of only the first 458 amino acids (Fig. 1c, cyan). We then performed coincubation assays in hydrogel using ES114 and the wild-type ES401 strain, or ES401 strains with mutations in *tasL* (*tasL* mutant), a T6SS2 structural protein (*tssF* mutant), or both (*tasL tssF* mutant). TssF is an essential baseplate protein (shown in yellow in Fig. 1a) that is necessary for T6SS function (21). Coincubation assays were performed as described previously (55, 59). Briefly, differentially tagged strains were mixed in a 1:1 ratio based on optical density and incubated in hydrogel or on agar surfaces for 12 h, and CFU were quantified for each strain type at the beginning and end of the experiment. CFU were then used to calculate the percent recovery of the competitor strain (ES114) after coincubation with the inhibitor strain.

Our coincubation results revealed a conditional role for TasL during T6SS-mediated competition. Consistent with our previous findings, the percent recovery of ES114 was significantly higher when it was incubated with the *tssF* mutant than with the wild type in hydrogel and on surfaces (Fig. 1d), indicating that a functional T6SS2 in ES401 is required to kill ES114 under both conditions. In hydrogel, the percent recovery of ES114 was not significantly different for coincubations with the *tasL* and *tssF* mutants (Fig. 1d), suggesting that TasL is required for killing in hydrogel. However, the percent recovery of ES114 in coincubations with the *tasL* mutant on surfaces was below 100 (indicating killing) and not statistically different from the percent recovery for coincubations with the ES401 wild type but significantly lower than the ES114 percent recovery for coincubations with the *tssF* mutant (Fig. 1d), suggesting that TasL is not required for killing on surfaces where contact between competing cell types is forced. Interestingly, there was a difference in effect size for the percent recovery of ES114 coincubated with the ES401 wild type and with the *tasL* mutant on surfaces (Fig. 1d). Although this difference was not statistically significant, these findings suggest that TasL may enhance T6SS-mediated competition when contact is forced. Furthermore, the percent recovery of ES114 was not significantly different for coincubations with the *tssF* mutant and the *tssF tasL* double mutant under both conditions (Fig. 1d), suggesting that the functions of these gene products are epistatic: the *tasL*-dependent phenotype was observed only in the presence of a functional *tssF* (or T6SS2). Finally, the *tasL* mutant grew to cell densities similar to those of the wild-type parent (Fig. S1a), and the *tasL* mutant was still able to build T6SS2 sheaths in hydrogel (Fig. S1b), suggesting that the *tasL* mutation does not impact growth or sheath assembly. Taken together, these data indicate that TasL is required for T6SS-mediated killing in hydrogel yet not on surfaces where contact is forced, which supports a model whereby TasL facilitates the cell-cell contact required for T6SS competition.

10.1128/mBio.03085-21.1 FIG S1 CFU for V. fischeri monocultures in hydrogel. (a) CFU quantified for V. fischeri monocultures from aggregation experiments in hydrogel. Strain genotype is indicated by the following: GI-, strain that does not carry the T6SS2 genomic island or *tasL*; WT, wild-type strain that carries *tasL*; mt, T6SS2^+^ strain that has a disruption mutation in *tasL*. Each experiment was performed twice with two biological replicates (*n* = 4). Error bars indicate SEM for combined experiments. (b) Fluorescence microscopy image of ES401 *tasL* mutant with VipA-GFP expression vector grown in hydrogel. White arrows indicate T6SS sheaths. Download FIG S1, EPS file, 0.1 MB. Copyright © 2022 Speare et al. 2022 Speare et al. https://creativecommons.org/licenses/by/4.0/ This content is distributed under the terms of the Creative Commons Attribution 4.0 International license.

### TasL is required for T6SS2-dependent phenotypes in the host.

Given that *tasL* is required for T6SS2-mediated competition under host-like conditions (hydrogel), we predicted that this protein may also affect competitive outcomes during host colonization. *E. scolopes* squid harbor multiple strains of V. fischeri in a structure called the light organ (60) that contains six independently colonized crypt spaces. Juvenile *E. scolopes* squid hatch with an aposymbiotic light organ that is colonized within hours by V. fischeri from the surrounding environment (61), allowing researchers to reconstitute the symbiosis in a lab setting. We previously showed that V. fischeri uses T6SS2 to prevent incompatible strains from coexisting within the same crypt space, thereby shaping the diversity and spatial structure of the microbial populations within a natural host (21). Furthermore, this T6SS-mediated competition can be visualized and quantified in the host by exposing juvenile squid to differentially tagged V. fischeri strains (21).

Before determining the ability of the ES401 *tasL* mutant to compete with ES114 during host colonization, we first tested the extent to which ES401 mutant strains (*tasL*, *tssF*, and *tasL tssF* mutants) are able to clonally colonize juvenile *E. scolopes* squid to ensure that their symbiotic competency is not different from that of the ES401 parent strain. These experiments are important to determine whether any of our ES401 mutants have a general colonization defect that could complicate the interpretation of our competitive colonization assays. After a 6-h exposure to each clonal inoculum, juvenile squid were transferred to bacterium-free water, where they remained for an additional 18 h to allow the animals to become fully colonized. At 24 h postinoculation, squid were measured for luminescence and CFUs were collected to determine the colonization ability of each ES401 strain. We found that each mutant achieved levels of luminescence and CFUs similar to those of the ES401 wild type (Fig. S2a and b), indicating that TasL and T6SS2 do not impact the ability of ES401 to colonize the squid light organ and luminesce.

10.1128/mBio.03085-21.2 FIG S2 TasL and T6SS2 do not impact the ability of ES401 to clonally colonize the squid light organ. Results from squid colonization experiments where juvenile *E. scolopes* squid were exposed to monocultures wild-type (WT) ES401 or *tasL*, *tssF*, or *tasL tssF* mutant strains with the inoculation ranging from 11,040 to 15,280 CFU/mL. Squid were exposed to the inoculum for 6 h, rinsed in filter-sterilized Instant Ocean, and incubated for an additional 15 h. At 24 h, squid were measured for luminescence, euthanized, and frozen at −80°C. CFU were collected by homogenizing each animal and plating on LBS agar plates within 48 h of freezing. The ability for each strain to colonize was assessed by examining (a) the number of CFU from each squid and (b) the amount of luminescence per squid. Each data point indicates results for a single animal. White data points indicate the limit of detection. Each experiment was performed once with 29 or 30 squid; all data are shown. Error bars indicate SD. Download FIG S2, EPS file, 0.2 MB. Copyright © 2022 Speare et al. 2022 Speare et al. https://creativecommons.org/licenses/by/4.0/ This content is distributed under the terms of the Creative Commons Attribution 4.0 International license.

We next determined the impact of TasL on competition during host colonization by quantifying two metrics: (i) the relative abundance of competing strain types in the host and (ii) the spatial distribution of strains in the light organ. The relative abundance of strains was determined by exposing juvenile squid to a 1:1 mixture of differentially tagged ES114 and ES401 wild-type and mutant strains for 9 h and determining the log relative competitive index (RCI) using CFUs collected from the mixed inoculum and from each squid 24 h after initial exposure. The spatial distribution of strains was determined by imaging the light organ using a fluorescence microscope to observe whether each colonized crypt contained a single strain (ES114 or ES401) or both strains, indicating that the crypt was cocolonized.

**FIG 2:**
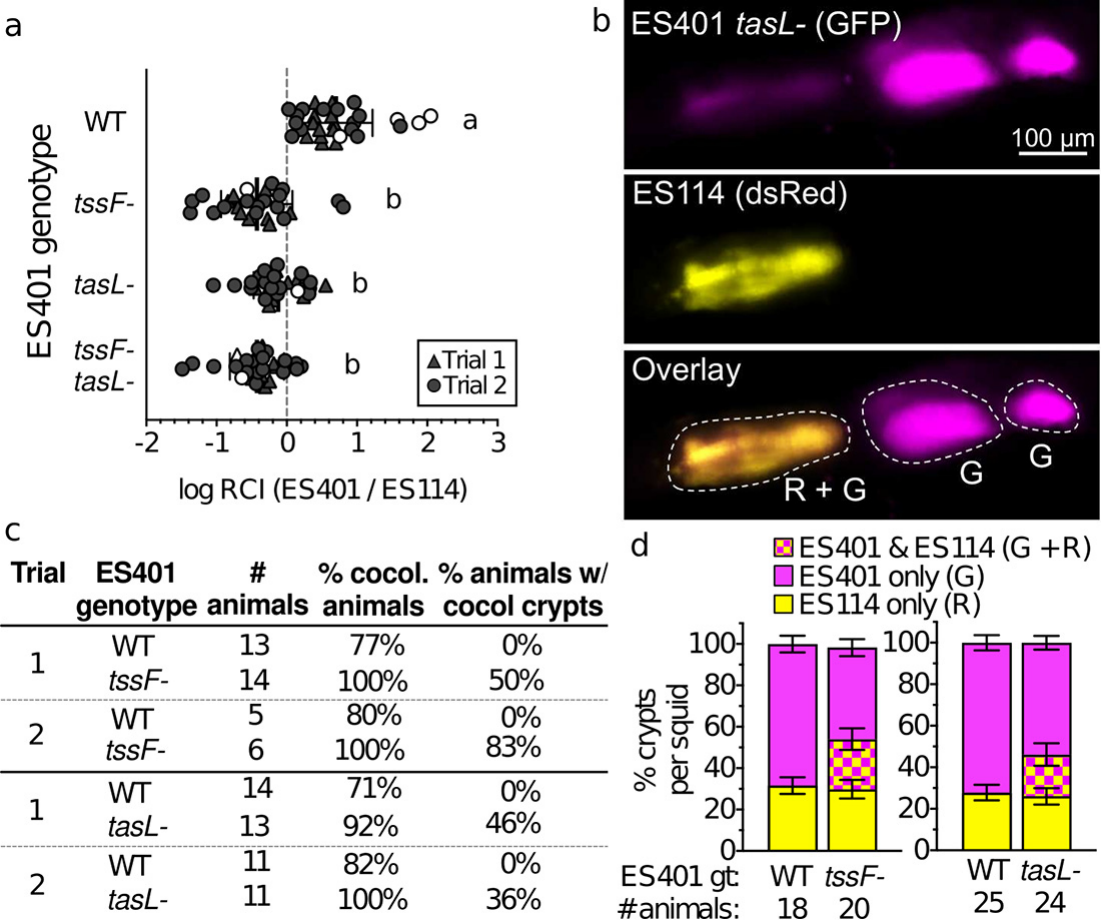
TasL is required for T6SS2-dependent phenotypes in the host. Results of squid colonization assays with strains ES114 and ES401 calculated from CFUs (a) or fluorescence microscopy images (b to d). (a) Log relative competitive index (RCI) values calculated for competition assays between ES114 and ES401-derived strains. Log RCI values were calculated by a ES401/ES114 ratio from CFUs collected at 24 h after initial exposure to inoculum divided by the ratio of these strains in the inoculum (starting ratio of ∼1:1). Each data point represents the log RCI value for an individual squid; the shape of each symbol indicates the trial in which the experiment was performed (triangle, trial 1; circle, trial 2), and white symbols indicate that a squid was colonized by only one strain type (<5% of squid in these trials). Letters indicate significantly different log RCI values between treatments (one-way ANOVA with Tukey’s multiple-comparison posttest: *P* < 0.01). Each experiment was performed twice; combined data are shown (*n* = 143). (b) Representative fluorescence microscopy images of a light organ colonized by differentially tagged ES401 *tasL* mutant (GFP tagged [G], shown here as magenta; top) and ES114 (DsRed-tagged [R], shown here as yellow; middle); an overlay of the two images is shown in the third row. Crypts were scored based on the presence of one or both strain types; crypts that contained only GFP (ES401) or only DsRed (ES114) were marked as singly colonized, and crypts containing both green and red fluorescence (ES401 and ES114) were marked as cocolonized. In the representative image, three crypts (outlined in white) were colonized: two singly colonized by the ES401 *tasL* (G) mutant and one cocolonized by the ES401 *tasL* mutant and ES114 (G+R). (c) Percentages of cocolonized animals and percentage of animals containing cocolonized crypts calculated from fluorescence microscopy images. (d) Percentages of crypts per squid containing only green fluorescence (ES401; magenta), only red fluorescence (ES114; yellow), or both green and red fluorescence (ES401 and ES114; checkered), calculated from data in panel c. For panels c and d, two trials of each experiment were performed; combined data are shown (total *n* = 87); error bars indicate standard errors of the means (SEM).

The results of our host colonization assays were consistent with the findings for the *in vitro* competition assays presented above. In the experiments measuring the relative abundance of each strain type, the log RCI values were significantly lower for competitive colonization assays using any of the three ES401 mutant strains than for the wild type, with an average effect size of ∼8-fold between the wild-type and mutant treatments (Fig. 2a). These data suggest that TasL is active within the natural host and indicate that both TasL and T6SS2 enhance the ability of ES401 to compete with ES114 during host colonization. Furthermore, log RCI data from host colonization assays reflect a similar outcome observed using coincubation assays in hydrogel (Fig. 1d), supporting the use of hydrogel as a tractable model to probe for competitive mechanisms that are active in the host yet are difficult to observe using surface-based culture conditions.

To determine how TasL impacts the spatial separation of strain types within the light organ, we performed cocolonization assays with differentially tagged ES114- and ES401-derived strains and used fluorescence microscopy to determine which strain type was in each colonized crypt. We hypothesized that if *tasL* is required for T6SS2-dependent strain separation in the host, then cocolonized crypts will be observed only for treatments where ES401 is impaired in its ability to engage in T6SS2- and *tasL*-dependent competition. Although the majority of animals were colonized by both strain types (71% to 100%), no cocolonized crypts were observed in experiments with wild-type ES401 (Fig. 2c). In contrast, 50 to 83% of animals had cocolonized crypts in experiments with the *tssF* mutant (Fig. 2c). This observation is consistent with previous findings that T6SS2 is required to spatially separate competing strain types during symbiosis establishment (21). In experiments with the *tasL* mutant, 36 to 46% of animals had cocolonized crypts (Fig. 2b and c), indicating that *tasL* is also required to prevent incompatible strain types from cooccupying the same crypt space. Finally, when we scored the proportion of crypts in each animal that were colonized by one or both strain types, we saw that the proportion of crypts colonized by ES114 alone did not change across treatments, while the proportion of crypts colonized by ES401 alone decreased with increasing crypt cocolonization frequency (Fig. 2d). Taken together, these data suggest that TasL and T6SS2 provide a competitive advantage during host colonization, such that in their absence ES401 does not eliminate ES114 in cocolonized crypts, resulting in loss of spatial separation of strain types and an increase in abundance of ES114, relative to ES401.

### TasL enhances cell-cell contact in hydrogel.

**FIG 3:**
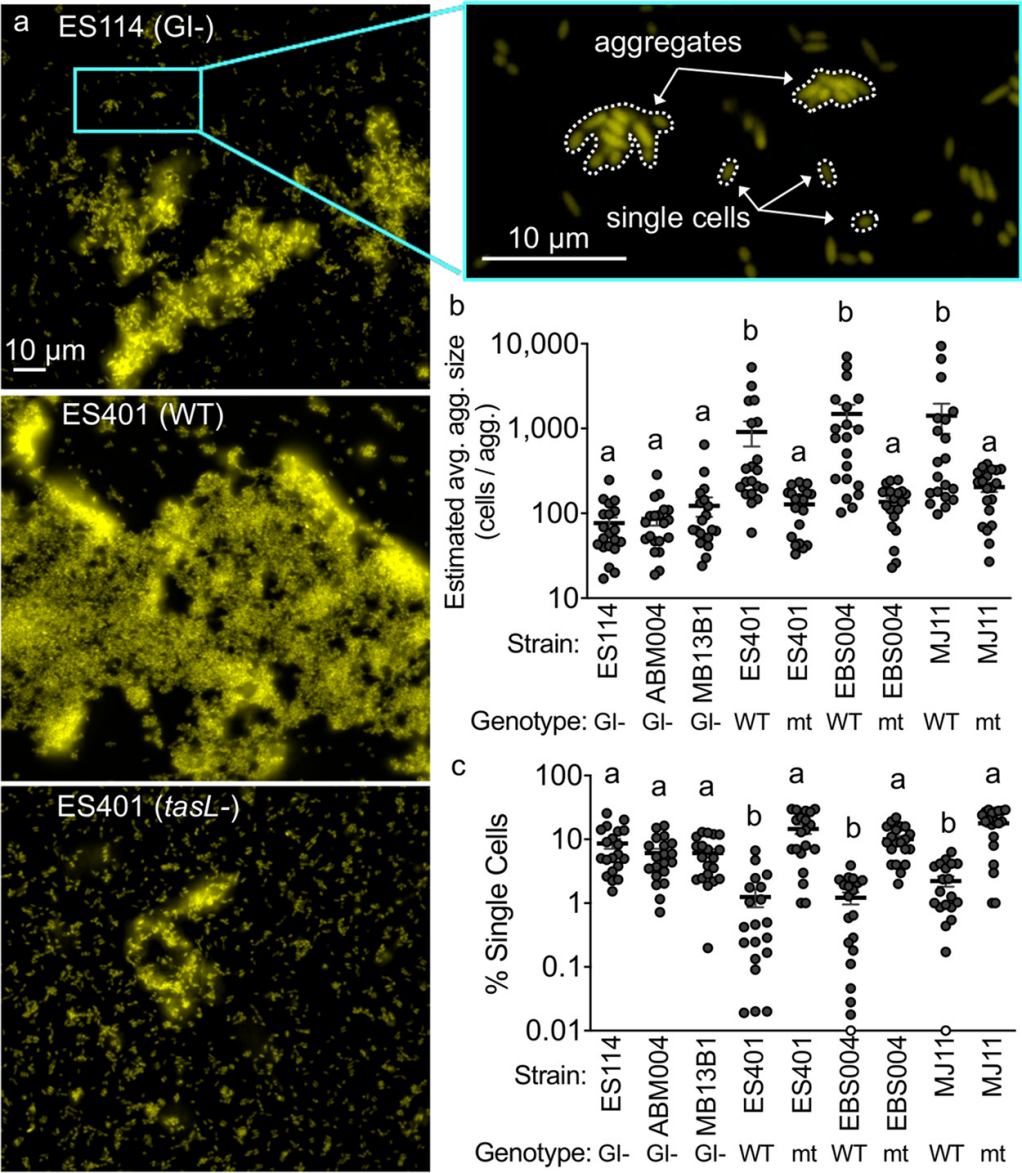
The presence of TasL correlates with greater cell-cell contact. (a) Representative single-cell fluorescence microscopy images of monocultures of V. fischeri strains and magnified image of representative ES114 image. Strain genotype is indicated as follows: GI-, strain that does not carry the T6SS2 genomic island or *tasL*; WT, wild-type strain that carries *tasL*; mt, strain that has a disruption mutation in *tasL*. Examples of single cells and cells in aggregates are outlined in the magnified image of ES114. (b) Estimated average aggregate size and (c) percentage of single cells for monocultures of each V. fischeri strain. Letters indicate significantly different estimated average aggregate size (b) or percent of single cells (c) between strains (two-way ANOVA with Tukey’s multiple-comparison posttest: *P* < 0.001). Each experiment was performed twice with two biological replicates and five fields of view; combined data are shown (*n* = 20). Error bars indicate SEM.

The findings from our assays above indicate that TasL is required for T6SS2-mediated killing under conditions where inhibitor cells must mediate contact with competitor cells, including in the host. To directly test whether TasL promotes cell-cell contact in hydrogel, we first determined the extent to which TasL affected aggregate size in monoculture. We took advantage of the natural strain-specific occurrence of the genomic island that encodes T6SS2 and TasL and selected three V. fischeri strains that do not contain the genomic island (ES114, ABM004, and MB13B1) and three strains that contain the genomic island (ES401, EBS004, and MJ11). We visualized monocultures of fluorescently tagged wild-type and *tasL* mutant strains grown in hydrogel and quantified aggregation ability based on two parameters: estimated average aggregate size and proportion of cells not in aggregates (percent single cells), as described in reference 62. Cells within each field of view were classified as single cells (two or fewer cells touching) or aggregated cells (three or more cells touching) based on the area of each particle and the average area of a V. fischeri cell (1.5 μm^2^). Consistent with our prediction that TasL promotes aggregation, the ES401 *tasL* mutant was unable to make large aggregates, compared to the wild type (Fig. 3a). Moreover, when we quantified the aggregation abilities of different strain types, we found that strains naturally lacking *tasL* or with a *tasL* disruption made significantly smaller aggregates (∼100 cells/aggregate) than strains encoding a functional TasL (∼1,000 cells/aggregate) (Fig. 3b). Taken together, these results suggest that TasL enhances aggregation ability in multiple V. fischeri strains.

We next aimed to quantify the extent to which TasL enhanced cell-cell contact. Given that the estimated cell counts were similar across strain types (Fig. S1a), we reasoned that quantifying the proportion of cells that are not able to integrate into aggregates (percent single cells) would be a good proxy for overall cell-cell contact. If a genotype is less efficient at aggregation, then we expect a larger proportion of single cells in that treatment. We found that strains that lack *tasL* or have a *tasL* disruption had a significantly higher percentage of single cells in culture than strains with a wild-type *tasL* gene (Fig. 3c), suggesting that TasL reduces the proportion of single cells in culture by promoting integration of cells into aggregates.

### TasL functions in a heterotypic manner to enhance cell-cell contact in hydrogel.

We next sought to determine whether TasL facilitates contact through a heterotypic interaction, such that *tasL* is required to be present in only one strain type, or a homotypic interaction, where *tasL* is required in both strain types. To distinguish between these possibilities, we performed pairwise aggregation assays with differentially tagged wild-type and *tasL* mutant strains and quantified aggregate size and the percentage of single cells in each treatment. If *tasL*-dependent aggregation is homotypic, then we expect to see large aggregates composed primarily of wild-type cells with *tasL* mutant cells composing the majority of the single-cell fraction. However, if *tasL*-dependent aggregation is heterotypic, then we expect to see well-mixed aggregates containing both wild-type and *tasL* mutant cells with a small proportion of single cells.

When we visualized cocultures of differentially tagged wild-type and *tasL* mutant strains, we observed aggregates containing both strain types, similar to what we observed for differentially tagged wild-type strains (Fig. S3a). Moreover, the estimated average aggregate size (Fig. S3b) and the percent single cells (Fig. S3c) were not significantly different between wild-type–*tasL* cocultures and differentially tagged wild-type cocultures. A significant difference in aggregation ability was observed only in the treatment where both strain types lacked *tasL*: the average estimated aggregate size was significantly smaller (Fig. S3b) and the percent single cells was significantly larger (Fig. S3c) than in the other treatments. These data indicate that *tasL* is required in only one strain type to enhance cell-cell contact in hydrogel and are consistent with a model whereby TasL promotes contact with competitor cells that do not carry *tasL*, such as ES114.

10.1128/mBio.03085-21.3 FIG S3 TasL promotes cell-cell contact in a heterotypic manner. (a) Representative single-cell fluorescent microscopy images for pairwise cocultures of differentially tagged ES401 wild-type (WT) and *tasL* mutant (*tasL-*) strains. Strain 1 (magenta) is shown in the first column, strain 2 (yellow) is shown in the second column, and an overlay is shown in the third column. (b) Average estimated aggregate size for each treatment. Letters indicate significantly different estimated average aggregate sizes between treatments (one-way ANOVA; Sidak’s multiple-comparison test: *P* < 0.0001). (c) Percent of single cells for each treatment. Letters indicate significantly different percentages of single cells between treatments (one-way ANOVA; Sidak’s multiple-comparison test: *P* < 0.0001). Each experiment was performed twice with two biological replicates and five fields of view; combined data are shown (*n* = 20). Error bars indicate SEM. Download FIG S3, EPS file, 0.9 MB. Copyright © 2022 Speare et al. 2022 Speare et al. https://creativecommons.org/licenses/by/4.0/ This content is distributed under the terms of the Creative Commons Attribution 4.0 International license.

### TasL promotes ES401-ES114 cell-cell contact in hydrogel.

**FIG 4:**
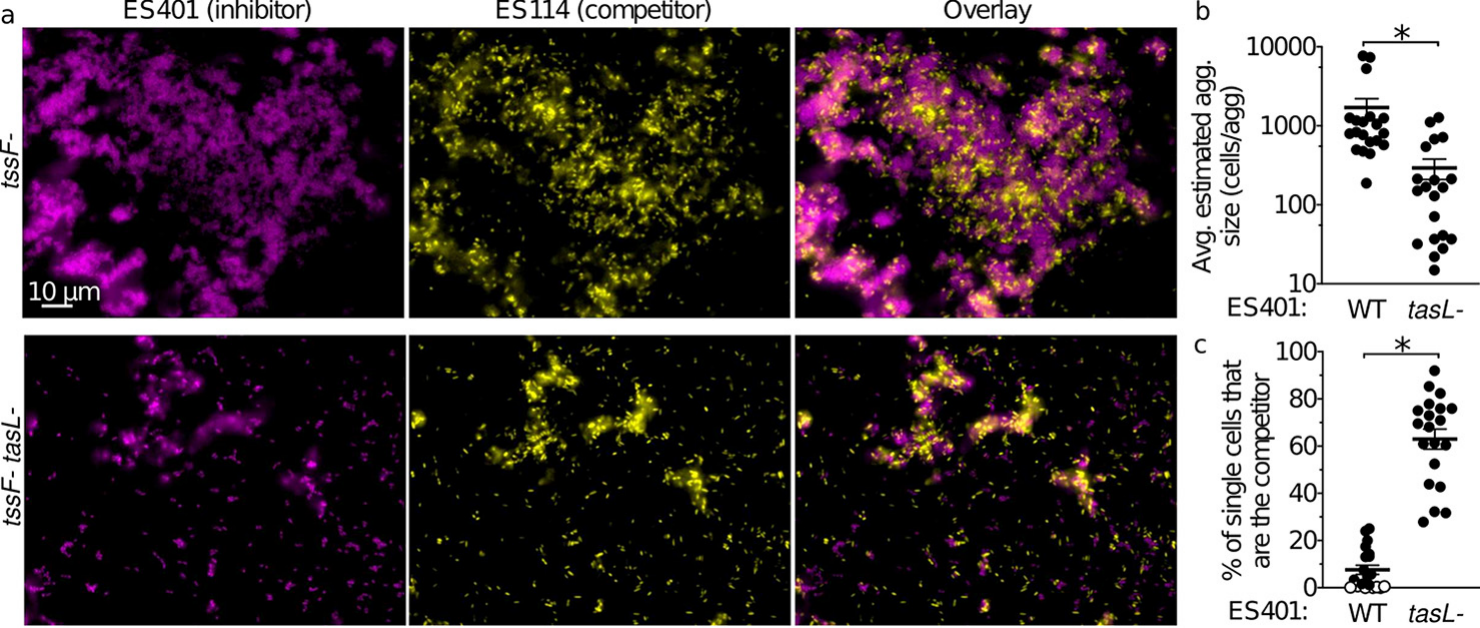
TasL promotes competitor-inhibitor cell-cell contact in hydrogel. (a) Representative fluorescence microscopy images of cocultures of the competitor strain ES114 (yellow) and ES401 *tssF_2* mutant (*tssF*-) or *tssF_2 tasL* double mutant (*tssF- tasL-*) strains (magenta). An overlay of the images of ES114 and ES401 is shown in the right column. The average estimated aggregate size (b) and percentage of single cells that are the competitor strain (ES114) (c) for experiments with ES114 and ES401 wild-type (WT) or *tasL* mutant (*tasL-*). Open circles indicate that <1% of single cells were the competitor strain. Asterisks indicate significantly different estimated average aggregate sizes (b) or percentages of single cells that are the competitor strain (c) (Student’s *t* test; *P* < 0.001). Each experiment was performed twice with two biological replicates and five fields of view; combined data are shown (*n* = 20). Error bars indicate SEM.

To directly test whether *tasL* is required for contact between inhibitor and competitor cells in hydrogel, we first visualized cocultures of ES114 (yellow) mixed with ES401 *tssF* strains containing wild-type or mutant *tasL* alleles (magenta). We chose to use T6SS2 (*tssF*) mutants to avoid the complication of inhibitor cells eliminating ES114 during the experiment. We predicted that if *tasL* is required for integration of ES114 competitor cells into ES401 aggregates in hydrogel, then there will be smaller aggregates with a higher proportion of cells, particularly ES114, that are located outside aggregates in cocultures using the *tasL* mutant relative to cocultures with the wild-type *tasL* allele. Although aggregates were observed in both treatments (Fig. 4a), they were significantly smaller in treatments with cells lacking a functional *tasL* (Fig. 4b). To specifically quantify the amount of ES401-ES114 contact in each treatment, we calculated the percentage of single cells that were ES114 for cocultures with ES401 wild-type and *tasL* mutant strains. ES114 cells made up a significantly larger proportion of the single-cell population in coincubations with the ES401 *tasL* mutant (∼60%) than the wild type (∼10%) (Fig. 4c). Taken together, these data indicate that *tasL* is necessary for mediating the cell-cell contact between inhibitor and competitor cells that is required for T6SS killing in hydrogel.

### TasL is necessary to discriminate between strain types in hydrogel.

**FIG 5:**
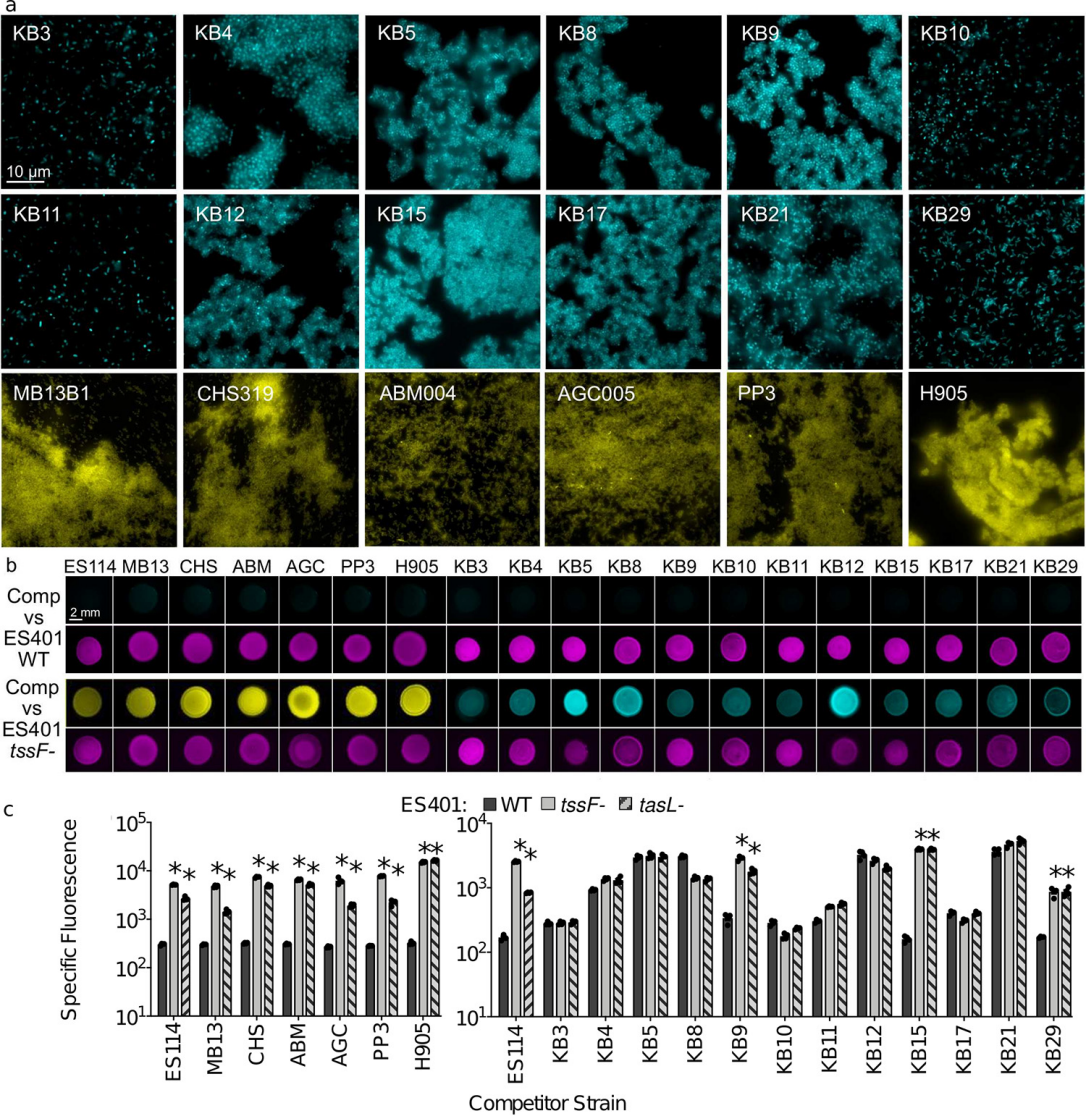
Only a subset of strains that are susceptible to T6SS2 killing on surfaces are also killed in a T6SS2- or *tasL*-dependent manner in hydrogel. (a) Representative fluorescence microscopy images of monocultures of competitor strains (strain name in upper left corner). KB isolates are shown in cyan, and LO isolates are shown in yellow. Each experiment was performed once with two biological replicates and five fields of view. (b) Fluorescent microscopy images from 24-h coincubation assays between each competitor (Comp) strain (KB isolate, cyan; LO isolate, yellow) and either wild-type (WT) or *tssF_2* mutant (*tssF-*) ES401 (magenta) on agar plates. Bar = 2 mm. (c) Specific fluorescence (green relative fluorescence units/OD) for 24 h hydrogel coincubation assays between GFP-tagged competitor strains (*x* axis) and wild-type (WT, dark gray), *tssF_2* mutant (T6S-, light gray), or *tasL* mutant (*tasL-*, hatched) ES401. Asterisks indicate experiments where specific fluorescence was significantly higher with the *tssF_2* and *tasL* mutants than the wild-type (two-way ANOVA with Dunnett’s multiple-comparison posttest; *P* < 0.0001). Abbreviations for longer competitor strain names are as follows: MB13, MB13B1; CHS, CHS319; ABM, ABM004; AGC, AGC005. Each experiment was performed three times, and results of one representative experiment are shown (*n* = 4). Error bars indicate standard deviations (SD).

**FIG 6:**
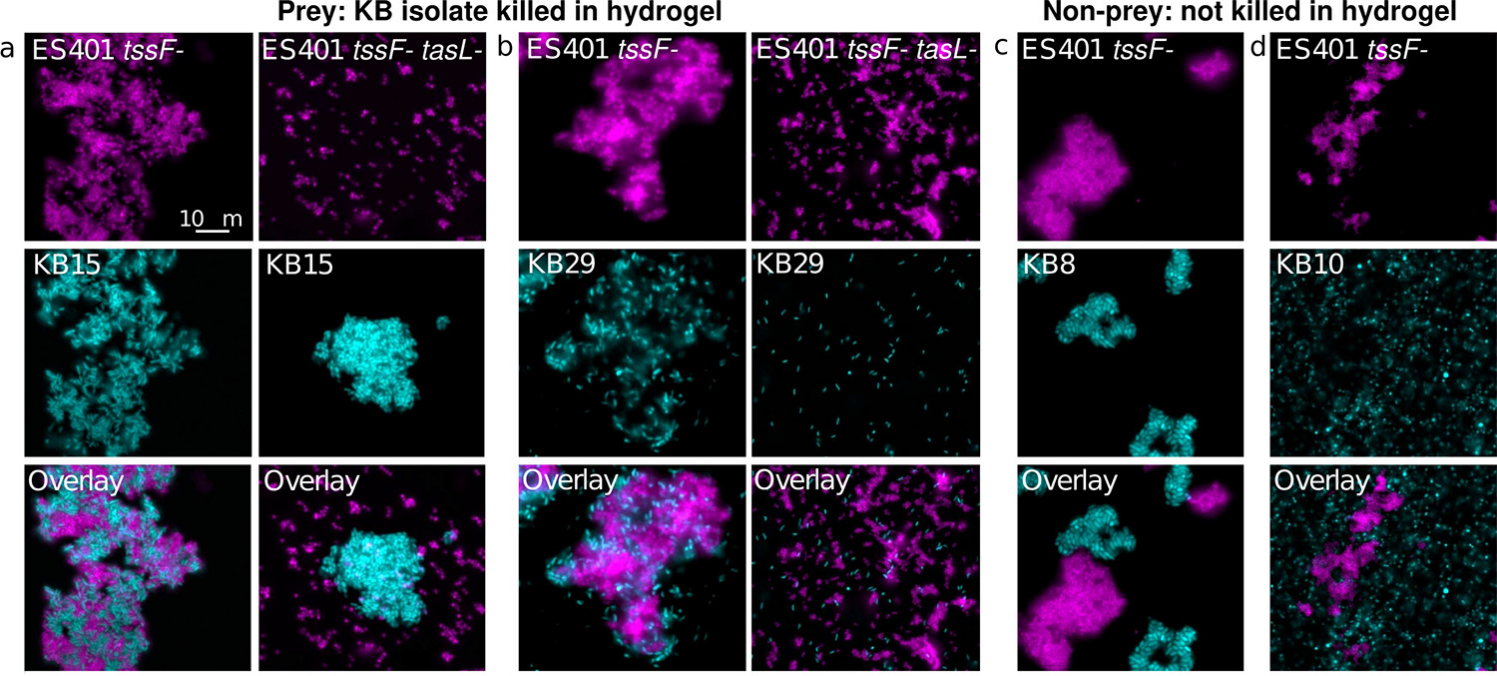
Nonprey strains avoid killing by ES401 in hydrogel through reduced cell-cell contact. Representative fluorescence microscopy images of cocultures of ES401 *tssF* or *tssF tasL* mutant strains (magenta) incubated with a KB isolate (cyan) after 12 h in hydrogel. ES401 strains were incubated with (a) KB15, (b) KB29, (c) KB8, or (d) KB10. KB15 and KB29 are prey (killed in hydrogel in a TssF- and TasL-dependent manner), while KB8 and KB10 are nonprey (not killed in hydrogel). Each experiment was performed twice with two biological replicates and five fields of view; one representative image is shown.

Given that *tasL* is required for contact with the competitor strain ES114, we wondered whether V. fischeri might require TasL to selectively target competitors of its ecological niche, the *E. scolopes* light organ. To explore this possibility, we selected 18 competitor strains: six additional *E. scolopes* light organ (LO) isolates and 12 *Vibrionaceae* isolates from Kaneohe Bay, HI (KB strains), where *E. scolopes* squid are endemic (Table S3). Because these strains’ inherent ability to aggregate in hydrogel may impact their capacity to be integrated into ES401 aggregates and therefore killed via T6SS2, we first assessed each strain’s aggregation ability by visualizing monocultures grown in hydrogel (Fig. 5a). Of the strains tested, all the LO isolates and the majority (8/12) of KB isolates could form aggregates in hydrogel. Moreover, all 18 strains were killed in a T6SS2-dependent manner on agar surfaces (Fig. 5b), indicating that they are susceptible to T6SS2 killing. However, KB isolates were less likely to be killed in a T6SS2- and *tasL*-dependent manner in hydrogel: only 25%, compared to 100% of light organ isolates (Fig. 5c). Importantly, there was no correlation between a KB strain’s abilities to make aggregates and resist killing in hydrogel, suggesting that KB cells can still be integrated into ES401 aggregates regardless of KB aggregation ability. When we directly imaged the coincubations between ES401 T6SS2 mutants with and without a functional *tasL*, we saw that KB strains that were outcompeted in hydrogel (prey strains) were integrated into ES401 aggregates in a *tasL*-dependent manner (Fig. 6a and b), while strains that were not outcompeted in hydrogel (nonprey strains) were not integrated into ES401 aggregates (Fig. 6c and d). Finally, when we mapped the prey/nonprey phenotype in hydrogel to strain phylogeny, based on *hsp60* sequences, we found prey strains throughout the tree, in both *Vibrio* and *Photobacterium* clades (Fig. S4), suggesting that *tasL*-dependent killing is not concordant with strain phylogeny. Taken together, these findings suggest that *tasL* is required for contact between ES401 and prey cells through a mechanism that is not restricted to V. fischeri and its closest relatives.

10.1128/mBio.03085-21.4 FIG S4 Sensitivity to killing in hydrogel is not correlated with phylogeny, and sensitive strains include both *Vibrio* and *Photobacterium* spp. Phylogenetic tree based on *hsp60* sequences for each target strain. Open circles indicate resistant strains, and closed circles indicate sensitive strains; strains highlighted in blue are from the genus *Vibrio*, and strains highlighted in purple are from the genus *Photobacterium.* Node values were calculated by maximum-likelihood bootstrap values. Download FIG S4, EPS file, 0.3 MB. Copyright © 2022 Speare et al. 2022 Speare et al. https://creativecommons.org/licenses/by/4.0/ This content is distributed under the terms of the Creative Commons Attribution 4.0 International license.

10.1128/mBio.03085-21.8 TABLE S3 Strains, plasmids, and oligonucleotides. Download Table S3, PDF file, 0.2 MB. Copyright © 2022 Speare et al. 2022 Speare et al. https://creativecommons.org/licenses/by/4.0/ This content is distributed under the terms of the Creative Commons Attribution 4.0 International license.

### ES401 preferentially targets specific competitor strains in a mixed culture.

**FIG 7:**
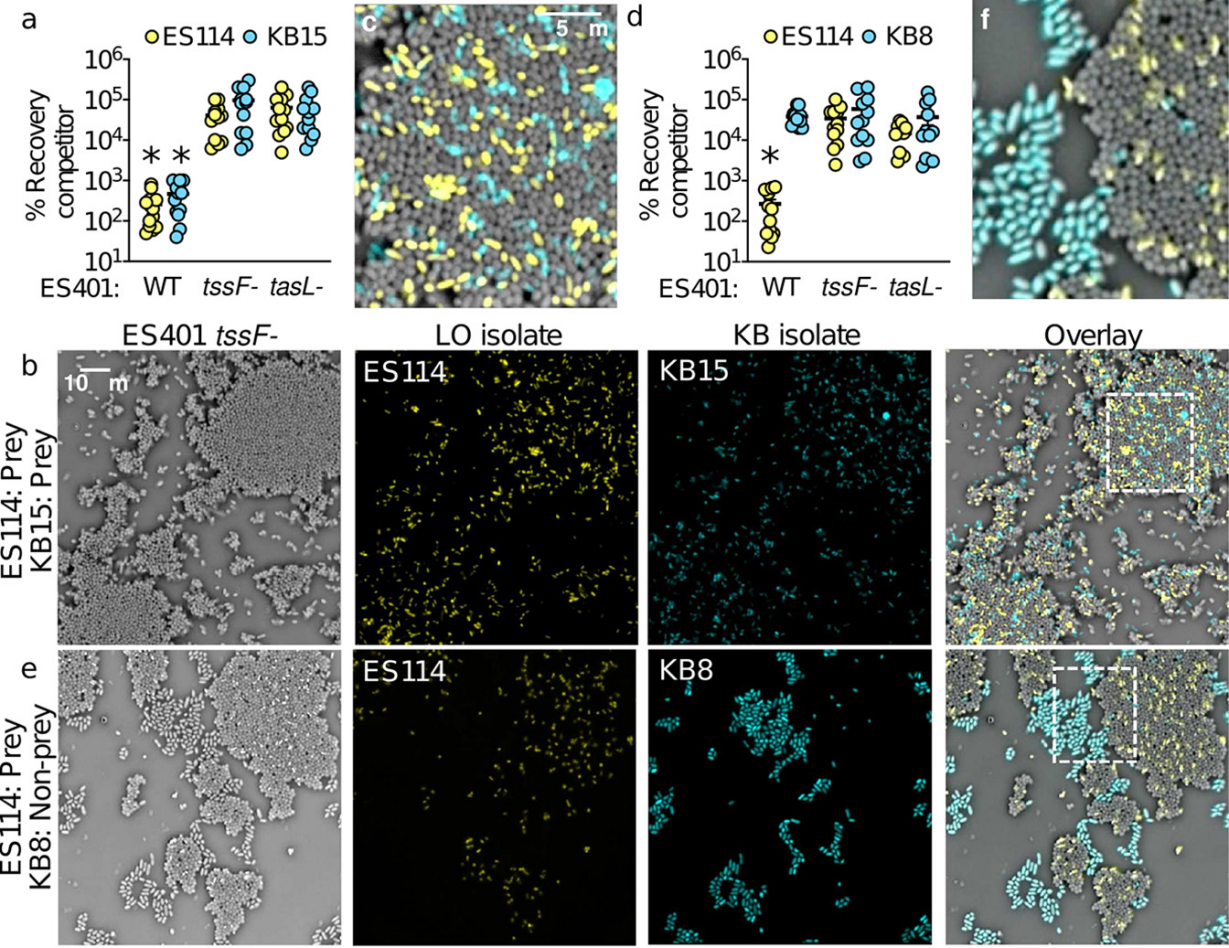
ES401 preferentially targets specific competitor strains in a mixed culture. (a to c) Results of 12-h coincubation assays in hydrogel between ES401 and two prey strains (killed in hydrogel): ES114 (yellow, LO isolate) and KB15 (cyan, KB isolate). (a) Percent recovery of each competitor strain. Asterisks indicate significantly lower percents recovery of a given competitor strain when incubated with wild-type ES401 relative to the *tasL* and *tssF* mutant strains (Student’s *t* test: *P* < 0.003). (b) Single-cell phase contrast and fluorescence microscopy images of coincubation assays in hydrogel with ES401 *tssF* (gray, column 1), ES114 (yellow, column 2), and KB15 (cyan, column 3); an overlay of all three images is shown in column 4. (c) Magnified view of the section of the overlay image outlined in panel b, column 4. (d to f) Results of 12-h coincubation assays in hydrogel between ES401 and one prey strain (killed in hydrogel; ES114, yellow, LO isolate) and one nonprey strain (not killed in hydrogel; KB8, cyan, KB isolate). (d) Percent recovery of each competitor strain. The asterisk indicates significantly lower percent recovery of a given target strain when incubated with ES401 wild-type than with the *tasL* and *tssF* mutant strains (Student’s *t* test; *P* < 0.003). (e) Single-cell phase-contrast and fluorescence microscopy images of coincubation assays in hydrogel with *tssF* ES401 (gray, column 1), ES114 (yellow, column 2), and KB8 (cyan, column 3); an overlay of all three images is shown in column 4. (f) Magnified view of the section of the overlay image outlined in panel e, column 4. Each experiment was performed three times, and either combined data (a and d: *n* = 12) or a representative image (b, c, e, and f: *n* = 1) is shown; error bars indicate SEM.

Our coincubation and aggregation assays indicate that *tasL* is required to make contact with specific competitor cells to facilitate T6SS2 killing in hydrogel. Given that ES401 likely encounters many cell types simultaneously in nature, we wondered whether ES401 would preferentially target competitors of the squid light organ (LO strains) in the presence of other cell types in the water column that would become enriched at the surface of the light organ through directed flow of seawater toward the pores (63, 64). To explore this possibility, we performed three-strain incubation assays in hydrogel by mixing equal numbers of ES401-derived strains with two different competitor strains: ES114 (LO isolate) and either KB15 (prey) or KB8 (nonprey). Because these KB isolates and ES114 do not prevent the growth of each other in coincubation assays on agar surfaces (Fig. S5), we can be confident that competitive outcomes observed in this experiment are the result of the ES401 genotype. In experiments with ES114 and KB15, the percent recovery of each competitor strain was significantly higher in incubations with the ES401 *tssF* or *tasL* disruption mutants than in incubations with the ES401 wild type (Fig. 7a), suggesting that both competitors were inhibited in a *tasL*- and T6SS2-dependent manner. When we visualized aggregates from these incubations, we observed that both competitor strains (ES114, yellow; KB15, cyan) were integrated into ES401 *tssF* mutant aggregates (gray) (Fig. 7b and c). However, in experiments with ES114 and KB8, the percent recovery of ES114 was significantly higher in incubations with the ES401 *tssF* or *tasL* mutants than with the wild type, while the percent recovery of KB8 was not significantly different between any treatments (Fig. 7d). Furthermore, while ES114 cells (yellow) were evenly distributed throughout ES401 *tssF* mutant aggregates (gray), very few KB8 cells (cyan) integrated into ES401 aggregates and KB8 cells instead formed spatially separated aggregates (Fig. 7e and f). Taken together, these data demonstrate that *tasL* is required for V. fischeri to preferentially contact specific competitor strains for T6SS2-mediated killing. Interestingly, ES401 did not preferentially target the LO isolate ES114 in the presence of strain KB15. This observation suggests that the mechanism by which *tasL* promotes target specificity is not strictly limited to competitors for the *E. scolopes* light organ and may be used by V. fischeri in locations outside the *E. scolopes* light organ crypts.

10.1128/mBio.03085-21.5 FIG S5 The KB isolates examined coexist with ES114 on agar surfaces. Fluorescence microscopy images from 24-h coincubation assays between KB isolates (cyan) and ES114 (yellow) on agar plates. Each KB isolate name is listed above its corresponding image. Each experiment was performed three times, and results of one representative experiment are shown. Download FIG S5, EPS file, 1.5 MB. Copyright © 2022 Speare et al. 2022 Speare et al. https://creativecommons.org/licenses/by/4.0/ This content is distributed under the terms of the Creative Commons Attribution 4.0 International license.

## DISCUSSION

This work demonstrates the use of a high-viscosity liquid medium (hydrogel) as a valuable model to examine bacterial behaviors that may be relevant in a host yet are difficult to study using traditional culturing methods. Using this model, we identified a large putative lipoprotein (TasL) that is encoded on the T6SS2 genomic island and necessary for the cell-cell contact required for T6SS2-mediated competition *in vitro* and *in vivo* in a natural host. The role of a lipoprotein mediating cell-cell contact is not without precedent. For example, surface-exposed lipoproteins have been shown to promote biofilm formation (65–69), mediate adhesion to epithelial cells (70), and even allow selective outer membrane transfer with specific bacteria (71). Moreover, this work shows that *tasL* impacts target specificity between ecologically relevant competitors, revealing a mechanism whereby a TasL^+^ strain establishes contact with specific competitor cells to restrict T6SS activity.

However, it is important to note that characterizing the function of a large gene (>10 kb) that contains multiple repeat sequences presented two main challenges in this study: (i) the need to make a disruption mutation rather than a clean deletion and (ii) the inability to construct a complementation vector. Due to the large size of *tasL* and the increased difficulty of creating clean deletions in many T6SS^+^ strains, we chose to make a disruption mutation to eliminate *tasL* function in ES401. Although clean deletions of genes are preferred to making disruption mutations, which can sometimes have pleiotropic effects, our data indicate this is not the case for the *tasL* disruption mutant. First, the *tasL* gene is not in an operon, and the upstream gene is divergently transcribed, while the downstream gene is convergently transcribed (Fig. 1b). Thus, it is unlikely that a disruption mutation in *tasL* would have polar effects on neighboring genes. Furthermore, when we quantified aggregation ability (both aggregate size and proportion of single cells) for strains that naturally do not carry *tasL*, we found that they possess the same decreased aggregation ability as the strains carrying a *tasL* disruption mutation (Fig. 3b and c). These data suggest that the smaller aggregate size of the *tasL* mutant is due to the absence of the wild-type allele, rather than the presence of a remaining N-terminal sequence. Furthermore, the *tssF* disruption mutant still makes large aggregates (Fig. 4a), indicating that the presence of an integrated plasmid for gene disruption is not the cause of decreased aggregation in the *tasL* mutant. Indeed, we could find no other defects for the *tasL* disruption mutant outside aggregation in hydrogel and its role in T6SS-mediated killing, and therefore, we see no evidence of pleiotropic effects from the use of a disruption mutation. Finally, we were unable to successfully generate a *tasL* expression vector to complement our *tasL* disruption mutant and express *tasL* in strains that naturally lack *tasL*. Although doing so would have indeed strengthened our conclusions, the enormous size of the gene and its many repeat sequences prevented the successful use of traditional cloning or synthesis approaches.

**FIG 8:**
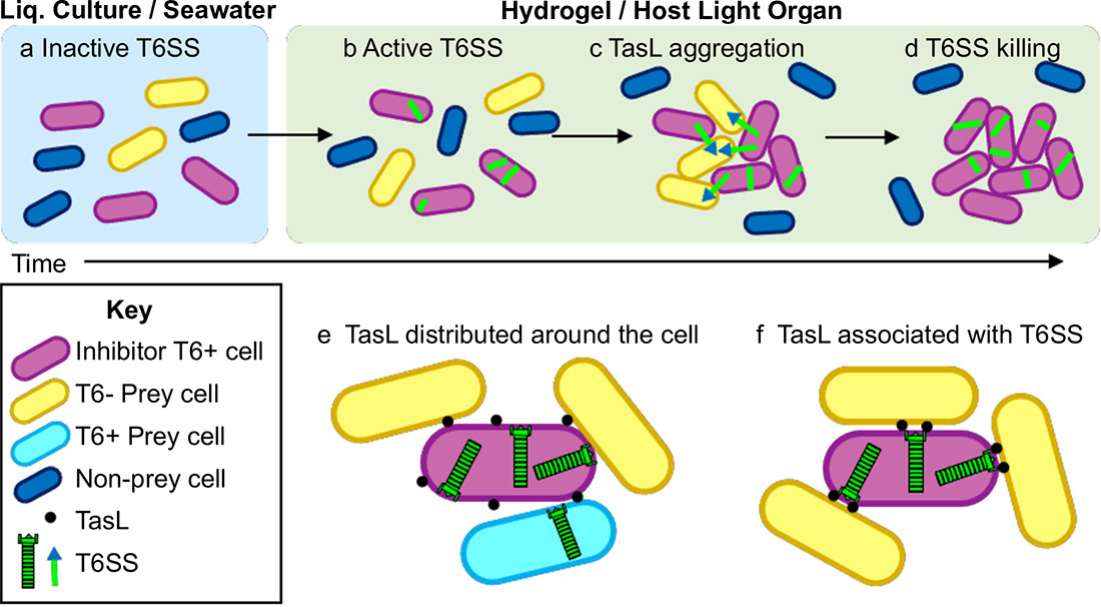
Predicted models for the coordination of TasL and T6SS for prey selection in hydrogel. Inhibitor cells encoding a functional T6SS (magenta, T6+) express TasL (black dots) and T6SS syringes (green), which are both necessary for establishing contact with and eliminating prey cells (yellow and cyan), while nonprey cells (dark blue) are excluded from aggregates and not killed. (a to d) Timeline for T6SS activity and TasL-mediated aggregation. (a) Cells in liquid (liq.) culture or seawater are physically dispersed, and T6SS/TasL is inactive. (b) As cells transition to hydrogel or become host associated, high-viscosity conditions promote T6SS and TasL expression and sheath assembly. (c and d) Cells then form TasL-mediated aggregates, which provides the necessary cell-cell contact for prey elimination. (e and f) Potential localization of TasL on inhibitor cells. (e) Random distribution of TasL on the inhibitor cell surface; (f) TasL colocalizing with T6SS structures.

Despite the technical challenges described above, our combined results suggest that TasL contributes to T6SS-mediated killing in a high-viscosity environment (i.e., hydrogel or the host light organ) through a three-step process. First, the transition from low to high viscosity activates T6SS2 and TasL expression in V. fischeri cells (55) (Fig. 8a and b). TasL then facilitates aggregate formation, whereby T6SS2^+^ TasL^+^ cells are in contact with prey (Fig. 8c). Finally, the T6SS2 syringe injects effectors into competitor cells within the aggregates, resulting in competitor elimination (Fig. 8d).

Based on our results and previous findings for the role of T6SS2 during juvenile *E. scolopes* colonization, we propose a model for how *tasL* contributes to T6SS-mediated competition during colonization of its natural host. Juvenile *E. scolopes* squid hatch with an aposymbiotic light organ, and planktonic V. fischeri cells transition from the seawater to the light organ surface, which is coated in a highly viscous mucus. After a few hours, V. fischeri cells migrate into the pores and through the ducts that lead to the crypt spaces. In some cases, crypts are initially cocolonized by cells of competing strain types. As these cells divide and grow, the populations make contact with one another and T6SS2^+^ strains eliminate competitor cells. Our data suggest that *tasL* is required for this T6SS phenotype in the host, likely through facilitating cell-cell contact between inhibitor and competitor (prey) cells as they fight for a preferred colonization site, resulting in crypts that are exclusively colonized by the T6SS2^+^ TasL^+^ genotype by 24 h.

Our observations raise questions from an ecological standpoint, as TasL-mediated target specificity is not limited to competitors for the squid light organ. TasL appears to promote elimination of a competitor within the squid light organ and also can selectively kill other water column strains that are unable to colonize *E. scolopes*. This observation suggests that TasL and T6SS2 may have an expanded role outside the *E. scolopes* light organ, such as in other eukaryotic hosts or under free-living conditions where T6SS2 and TasL are active (e.g., high viscosity habitats). Furthermore, given the prevalence of TasL homologs in other T6SS-encoding vibrios (Table S1), this work provides exciting evidence that coevolved competitors may employ large, putative lipoproteins to facilitate T6SS-mediated killing of specific competitors in a liquid environment.

Although this work did not identify the underlying molecular mechanism by which TasL promotes contact with prey cells, our data support the possibility that TasL may interact with a ligand displayed on the cell surface of specific genotypes, similar to the receptor-ligand function of phage tail fibers and RBPs. For nonprey KB isolates, such a ligand may be absent, or the ligand could be present but obscured by other cell surface structures that prevent interactions with TasL. Similar receptor modification/occlusion has been reported for bacterium-phage interactions (72), where bacteria can modify surface receptors that are recognized by phage through posttranslational modifications (via glycosylation) (73) or by masking/hiding surface receptors (74, 75). Such a mechanism could explain the strain-specific variation in aggregation among KB isolates, which may be a result of the mechanistic interactions between TasL and its receptor.

Further examination of the nature of TasL-dependent cell contact, as well as its localization, will provide important insight into how V. fischeri has evolved mechanisms to restrict T6SS-mediated killing to compete for the host niche. For example, if TasL is indeed localized to the outer membrane, then based on our findings, we propose two possible models for how distribution of TasL on the cell surface may impact the survivability of bacterial cells during competition. We predict that TasL could be randomly distributed on the cell surface (Fig. 8e) or colocalized specifically with T6SS structures (Fig. 8f). Although a random distribution of TasL might increase the chances of contacting a competitor cell, if contact is made where a T6SS weapon is not found, the competitor will not be eliminated. Moreover, if the competitor cell that is brought into contact also has a T6SS, the inhibitor is at risk of being eliminated due to its own adhesion mechanism. Alternatively, if TasL colocalizes with the T6SS structure, similar to phage tail fibers and the baseplate (76), then all TasL-mediated contact will correspond to the ability to eliminate the competitor. Therefore, we hypothesize that selective pressure to survive such encounters would favor TasL colocalization with T6SS weapons.

## MATERIALS AND METHODS

See Text S1 for additional experimental details, including media and growth conditions, isolation of Kaneohe Bay bacteria, strain and plasmid construction, high-throughput modifications to coincubation assays, and phylogenetic analysis. Bacterial strains, plasmids, and oligonucleotides used in this study are listed in Table S3.

10.1128/mBio.03085-21.9 TEXT S1 Additional experimental details on media and growth conditions, isolation of Kaneohe Bay bacteria, strain and plasmid construction, high-throughput modifications to coincubation assays, and phylogenetic analysis. Download Text S1, DOCX file, 0.03 MB. Copyright © 2022 Speare et al. 2022 Speare et al. https://creativecommons.org/licenses/by/4.0/ This content is distributed under the terms of the Creative Commons Attribution 4.0 International license.

### Coincubation assay.

Coincubation assays on surfaces and in hydrogel were performed as described previously (21, 55, 59). Briefly, shaking overnight cultures of differentially tagged V. fischeri strains or KB isolates grown in Luria-Bertani salt (LBS) broth supplemented with the appropriate antibiotic at 24°C were diluted to an optical density at 600 nm (OD_600_) of 1.0. Strains were mixed in either a 1:1 or 1:5 ratio (based on OD), and 10 μL of the mixture was spotted into wells containing 1 mL hydrogel medium (LBS broth with 5% polyvinylpyrrolidone [PVP]) or onto LBS agar plates and incubated at 24°C without shaking. At indicated time points, strains in each coincubation were quantified by plating serial dilutions onto LBS plates supplemented with antibiotics selective for each strain.

### Squid colonization assays.

Overnight cultures of each strain were diluted 1/100 into artificial seawater-tryptone (ASWT) and grown to an OD_600_ of ∼0.5. For each set of squid colonization experiments, freshly hatched juvenile squid were exposed to the inoculum for 6 h (monocultures) or 9 h (competitions) and rinsed in fresh filter-sterilized Instant Ocean. At 24 h, animals were measured for luminescence using a Turner BioSystems 20/20^n^ luminometer, euthanized with 2% ethanol, and either plated for CFU or prepared for fluorescence microscopy.

For single-strain colonization experiments, 30 squid were exposed to a single-strain inoculum containing green fluorescent protein (GFP)-tagged ES401 wild-type, *tssF* mutant, tasL mutant, or tssF tasL mutant strains at a final concentration ranging from 11,040 to 15,280 CFU/mL. This experiment was performed once with 30 squid per treatment, resulting in a total of 120 animals. Two competitive colonization experiments were performed with 11 to 24 squid that were exposed to an even mix of differentially tagged ES114 and indicated ES401-derived strains at a final concentration ranging from 17,260 to 39,360 CFU/mL (*n* = 153). Competitive fitness is presented as log RCI, which was calculated by dividing the ratio of ES401 to ES114 in each squid by the initial ratio of ES401 to ES114 in the inoculum and taking the log of that value. Log RCI values were compared between treatments to determine the impact of ES401’s genotype on competitive outcomes. Squid colonization was quantified as described by Naughton and Mandel (77).

To determine the spatial distribution of genotypes within the squid light organ, 5 to 15 freshly hatched juvenile squid were exposed to an even mix of differentially tagged ES114 and ES401 strains at a final concentration ranging from 16,640 to 23,040 CFU/mL (21, 78). Animals were euthanized in 2% ethanol and prepared for fluorescence microscopy by dissecting the ventral side of the mantle and removing the siphon to reveal the light organ. Each light organ was imaged for green and red fluorescence with a 10×/1.3 numerical aperture oil Ph3 lens objective, and images were captured with an Olympus BX51 microscope outfitted with a Hamamatsu C8484-03G01 camera using MetaMorph software. Each crypt space was scored separately for green fluorescence (ES401) and red fluorescence (ES114); aposymbiotic squid were also imaged as controls. Four separate trials were performed with 5 to 15 squid (*n* = 87 squid). The laboratory practices were carried out using procedures approved by IACUC.

### Single-cell fluorescence microscopy.

Visualization and quantification of aggregates in hydrogel were performed as described previously (55, 62). Briefly, overnight cultures of V. fischeri or KB strains containing either pVSV102 (GFP) or pVSV208 (DsRed) were grown in LBS broth supplemented with the appropriate antibiotic at 24°C. Strains were normalized to an OD_600_ of 1.0, mixed in a 1:1 ratio, and incubated in hydrogel (LBS broth plus 5% PVP). After 12 h of incubation, 5 μL of culture was spotted directly onto a glass slide and imaged with a 60×/1.3 numerical aperture oil Ph3 lens objective. Images were captured with an Olympus BX51 microscope outfitted with a Hamamatsu C8484-03G01 camera using MetaMorph software. The estimated average aggregate size was determined by using the “image/adjust/threshold” and “analyze particles” commands to calculate the area of each particle. Particles larger than or equal to the area of three cells (4.5 μm^2^) were defined as aggregates, and particles less than the area of three cells were defined as single cells. The proportion of each strain type in the single cell fraction was calculated by quantifying the total area of the single-cell fraction in the composite image of both strains. Next, the proportion of the single-cell area that showed either green or red fluorescence was determined and divided by the total area of the single-cell fraction. To ensure that the green and red fluorescent proportions were accurately calculated, they were summed to confirm that they added up to 1.0 (the entire area of the single-cell fraction). Visualization of VipA-GFP sheaths was performed with cultures of ES401 carrying the IPTG (isopropyl-β-d-thiogalactopyranoside)-inducible VipA_2-GFP expression vector (pSNS119) as described previously (55, 62).
