# Supplementary material for: A Putative Lipoprotein Mediates Cell-Cell Contact for Type VI Secretion System-Dependent Killing of Specific Competitors
Source: mBio. 2022 Apr 11;13(2):e03085-21. doi: 10.1128/mbio.03085-21 (PMC9040878; doi:10.1128/mbio.03085-21)
Supplement: TABLE S1 [file mbio.03085-21-st001.pdf]

Table S1. Distribution of *V. fischeri* TasL proteins associated with bacterial species associated with a marine host. Percent identity based on BlastP results using VFES401_15750 (TasL) as sequence query.

**Bacterial species**	**VFES401_15750 homolog**	**% ID**
Vibrio fischeri ES401	TGA68331.1	100 %
V. fischeri MJ11	ACH64371.1	98 %
V. wodanis	WP_061004509.1	65 %
V. logei	WP_083199035.1	61 %
V. tubiashii	WP_052123152.1	36 %
V. campbellii	WP_103414330.1	35 %
V. harveyi	SUP42962.1	35 %
V. vulnificus	WP_166735628.1	35 %
V. alginolyticus	WP_158155857.1	34 %
V. parahaemolyticus	WP_153884732.1	34 %
V. owensii	WP_122045168.1	34 %
Moritella viscosa	SGY96550.1	33 %
