# Supplementary material for: A Putative Lipoprotein Mediates Cell-Cell Contact for Type VI Secretion System-Dependent Killing of Specific Competitors
Source: mBio. 2022 Apr 11;13(2):e03085-21. doi: 10.1128/mbio.03085-21 (PMC9040878; doi:10.1128/mbio.03085-21)
Supplement: TABLE S2 [file mbio.03085-21-st002.pdf]

Table S2. Abundance of large lipoproteins in close proximity to T6SS gene clusters.

Bacterial species	Protein ID (KEGG)	T6SS gene cluster
Xanthomonas citri pv. citri 306	XAC_4113	XAC_4112 – 4147
Dyella thiooxydans	ATSB10_15920	ATSB10_15900 - 16200
Myxococcus xanthus DK	MXAN_4798	MXAN_4800 - 4813
