# Supplementary material for: A Putative Lipoprotein Mediates Cell-Cell Contact for Type VI Secretion System-Dependent Killing of Specific Competitors
Source: mBio. 2022 Apr 11;13(2):e03085-21. doi: 10.1128/mbio.03085-21 (PMC9040878; doi:10.1128/mbio.03085-21)
Supplement: TABLE S3 [file mbio.03085-21-st003.pdf]

Table S3. Strains, plasmids, and Oligo table.

**Wild-type Vibrionaceae**	**Host / Source**	**Reference**
ABM004	*Euprymna scolopes* light organ	Speare et al 2018
AGC005	“”	“”
CHS319	“”	“”
EBS04	“”	“”
ES114	“”	Boettcher and Ruby 1994
ES401	“”	Lee 1994
H905	Kaneohe Bay water column isolate that colonizes *E. scolopes* light organ	Lee and Ruby 1992
KB3	Water column from Kaneohe Bay, HI<br>GPS coordinates: 21°25’44”N 157°47’33” W	This Study
KB4	“”	“”
KB5	“”	“”
KB8	“”	“”
KB9	“”	“”
KB10	“”	“”
KB11	“”	“”
KB12	“”	“”
KB15	“”	“”
KB17	“”	“”
KB21	“”	“”
KB29	“”	“”
MB13B1	*E. scolopes* light organ	Wollenberg and Ruby 2009
MJ11	*Moncentris japonica* light organ	Ruby and Nealson 1976
PP3	Kaneohe Bay water column isolate that colonizes *E. scolopes* light organ	Lee and Ruby 1992
**Strain**	**Relevant Characteristics**	**Reference**
ANS2100	*V. fischeri* strain ES401 with a disruption in *vasA_2* (Erm^R)	Viscosity MS
ANS2101	*V. fischeri* strain ES401 with a disruption in *tasL* (VFES401_15750) (Erm^R)	This Study
CC118λpir	*E. coli; ∆(ara-leu) araD ∆lac74 galE galK phoA20 thi-1 rpsE rpsB argE*(Am) *recA λpir*	Herrero et al., 1990
DH5α	*E. coli; F’/endA1 hsdR17 glnV44 thi-1 recA1 gyrA relA1 ∆(lacIZYAargF) U169deoR(f80dlacI* ∆(lacZ)*M15)*	Hanahan, 1983
DH5αλpir	*E. coli; λpir* derivative of DH5α	Dunn *et al.,* 2005
LAS013	ES401 with disruptions in *vasA_2* and *tasL* (Erm^R, Cm^R)	This Study
LAS014	EBS004 with a disruption in *tasL* (VFES401_15750) (Erm^R)	“”
LAS015	MJ11 with a disruption in *tasL* (VFES401_15750) (Erm^R)	“”
**Plasmids**	**Relevant Characteristics**	**Reference**
pAS2031	*tasL* disruption vector; $oriV_{R6K\gamma}$, *oriT*, Erm^R	This Study
pEVS104	conjugative helper, $oriV_{R6K\gamma}$, *oriT* , Kn^R	Stabb & Ruby, 2002
pEVS118	Suicide vector, $oriV_{R6K\gamma}$, $oriT_{RP4}$, Cm^R	Dunn *et al*., 2005
pLS05	*tasL* disruption vector, $oriV_{R6K\gamma}$, *oriT*, Cm^R	This Study
pVSV102	*gfp*+, $oriV_{R6K\gamma}$, $oriV_{pES213}$, *oriT*, Kn^R	Dunn *et al.,* 2006
pVSV122	Suicide vector, $oriV_{R6K\gamma}$, *oriT*, Erm^R	Dunn *et al*., 2005
pVSV208	*dsRed*+, $oriV_{R6K\gamma}$, $oriV_{pES213}$, *oriT*, Cm^R	Dunn *et al.,* 2006

**Oligonucleotide**		
AS1105	GAGCTCGGTACCCGGGGATCCGCCAATCGAA TAATGTTGACG	This Study
AS1106	CTCAAGCTTGCATGCCTGCAGGTGGCAGATT CAATTTCAACC	“”
LS015	GAGATCTACTAGTGGCCAGGTGCCAATCGAA TAATGTTGACG	“”
LS016	GTACTGCCTTCCAGTCTAGTTCTGGCAGATTC AATTTCAACC	“”
LS017	GAACTAGACTGGAAGGCAGTAC	“”
LS018	ACCTGGCCACTAGTAGATCTC	“”
LS021	GAGATCTACTAGTGGCCAGGTTGTTGATCCGA TTTCAGGTGG	“”
LS022	GTACTGCCTTCCAGTCTAGTTCTTAGTGGTGG TGGTGGTGGTGTGCAGCCTTTCTTGTTAACCA	“”
H279	GAATTCGANNNNGCNGGNGAYGGNACNACNA C	Goh *et al.*, 1996
H280	CGCGGGATCCYKNYKNTCNCCRAANCCNGGN GCYTT	“”
