# Supplementary material for: A Putative Lipoprotein Mediates Cell-Cell Contact for Type VI Secretion System-Dependent Killing of Specific Competitors
Source: mBio. 2022 Apr 11;13(2):e03085-21. doi: 10.1128/mbio.03085-21 (PMC9040878; doi:10.1128/mbio.03085-21)
Supplement: TEXT S1 [file mbio.03085-21-t0001.docx]

**Supplemental Methods**

**Media and growth conditions.** *V. fischeri* strains were grown in LBS medium [1] at 24°C and *E. coli* strains were grown in either LB medium [2] or Brain Heart Infusion (Difco) at 37°C. Antibiotic selection for *V. fischeri* and *E. coli* strains were as described previously [3]. Plasmids with the R6Kγ origin of replication were maintained in *E. coli* strain DH5αλpir [3] and plasmid pEVS104 [4] was maintained in strain CC118λpir [5]. All other plasmids were maintained in *E. coli* strain DH5α [6].

**Isolation of Kaneohe Bay bacteria**. In May 2016, 45 mL seawater samples were collected in 50 mL sterile capped conical tubes (VWR) from the water column at the shore of Kaneohe Bay (21°25’44”N 157°47’33” W) and transported back to the laboratory on ice. Thirty mL water samples were syringe-filtered onto 25 mm Supor® 200 0.2 mM pore size PES membrane disc filters (Pall Corporation) held in sterilized reusable 25 mm syringe filter holders (Pall Corporation). Filters were removed from the filter units using sterilized forceps and placed in sterile 2 mL screw-cap tubes containing 1 mL of filter-sterilized artificial seawater (35 ppm Instant Ocean®) with 20% final volume of glycerol. Samples were frozen at -80°C for three weeks until processed. Frozen tubes containing the filters were thawed on ice, gently agitated, and the filter and liquid was placed into a 50 mL sterile capped conical tube. One mL of sterile artificial seawater was used to rinse the filter and the tube was vortexed briefly to mix. One hundred mL aliquots were removed from the tube and plated onto Difco™ TCBS agar. Plates were incubated in the dark at 25°C, and colonies with distinct morphologies were restreaked onto TCBS agar after either one or two days of incubation. Isolates were restreaked at least two times to ensure that streaks did not contain more than one bacterial strain. Streak-purified isolates were grown overnight with shaking in LBS medium at 25°C and stocked as 20% final volume glycerol stocks at -80°C.

**Strain and plasmid construction.** Bacterial strains, plasmids, and oligonucleotides used in this

study are presented in Table S1. For mutant construction in *V. fischeri*, mutant alleles were mobilized on plasmids into recipients by triparental mating using CC118λpir pEVS104 as a

conjugative helper [3-5]. Potential mutants were screened for appropriate antibiotic resistance markers and verified using PCR. Primer design was based on either ES401 or MJ11 genome sequence. To construct the *tasL* disruption mutant, approximately 1 kb of the *tasL* gene was PCR amplified using primers AS1105 and AS1106 from *V. fischeri* strain ES12 gDNA. The suicide vector pES122 was amplified using primers AS1107 and AS1108. The resulting PCR reactions were digested with DpnI to remove any contaminating template DNA, cleaned and concentrated using a Zymo Clean/Concentrate kit, and combined using SLiCE [7], resulting in the *tasL* disruption construct, pAS2031. The *tasL* disruption construct on pAS2031 was moved into strain ES401, EBS004, and MJ11 resulting in strains ANS2101, LAS014, and LAS015, respectively.

A *tasL* disruption construct encoding chloramphenicol resistance, pLS005, was constructed to generate a *vasA_2* (*tssF_2*) *tasL* double disruption mutant. To construct pLS005, approximately 1 kb of the *tasL* gene was PCR amplified using primers LS015 and LS016 from ES401 gDNA. The resulting PCR product was cloned into the KpnI and SphI sites of plasmid pEVS118 using the standard sequence-and ligation-independent cloning (SLIC) technique [8]. The *tasL* disruption construct on pLS005 was moved into strain ANS2100 resulting in strain LAS013.

**High throughput Coincubation Assay.** A high throughput modification to the standard hydrogel coincubation assay was optimized to determine whether multiple competitor strains were susceptible to T6SS2- or *tasL-*dependent killing within the same experiment. Overnight cultures of GFP-tagged light organ (LO) isolate, Kaneohe Bay (KB) isolate, and untagged ES401-derived strains were diluted to an OD_600_ of 1.0. Each LO and KB isolate (GFP-tagged) was mixed with each ES401 strain in a 1:5 (competitor:ES401) ratio based on OD and 2 μl of each mixture was spotted into a well a 96-well plate containing 200 μl of hydrogel in each well and incubated at 24°C without shaking. At 24 h the specific fluorescence of each well was quantified and then divided by the total OD. Specific fluorescence / OD was compared between treatments to determine whether values were significantly higher for coincubations with the *tssF* or *tasL* mutant relative to the wild-type ES401, indicating T6SS2- and/or TasL-dependent killing had occurred. Coincubations between ES114 and ES401 strains were included as a positive control.

**Phylogenetic analysis.** A single locus analysis was performed using the *hsp60* gene sequence from published sequence data and newly amplified sequences of 14 total bacterial strains were collected and aligned with ClustalW [9]. Newly amplified sequences have been deposited in the GenBank database (accession numbers: pending). Phylogenetic constructions assuming a tree-like topology were created with maximum likelihood (ML), neighbor joining (NJ), and maximum parsimony (MP). ML and MP reconstructions were performed by treating gaps as missing and MP, MP, and NJ analyses likelihood scores of 1400+ potential evolutionary models were evaluated using the corrected Akaike Information Criterion and Baysian Information Criterion. The most optimal evolutionary model for each evaluation was the Kimura 2-parameter model and a discrete Gamma distribution to model evolutionary rate differences among sites (K2+G). Evolutionary analyses were conducted in MEGA X [10]. Phylogenetic trees were visualized with MEGA X and the final tree was edited for publication with Inkscape 1.0 (http://inkscape.org/).

**References**

1. Stabb, E.V., K.A. Reich, and E.G. Ruby, *Vibrio fischeri Genes hvnA andhvnB Encode Secreted NAD+-Glycohydrolases.* Journal of Bacteriology, 2001. **183**(1): p. 309-317.

2. Miller, J., et al., *A short course.* Bacterial Genetics, 1992.

3. Dunn, A.K., M.O. Martin, and E.V. Stabb, *Characterization of pES213, a small mobilizable plasmid from Vibrio fischeri.* Plasmid, 2005. **54**(2): p. 114-134.

4. Stabb, E.V. and E.G. Ruby, *RP4-based plasmids for conjugation between Escherichia coli and members of the Vibrionaceae.* Methods Enzymol., 2002. **358**.

5. Herrero, M., V. de Lorenzo, and K.N. Timmis, *Transposon vectors containing non-antibiotic resistance selection markers for cloning and stable chromosomal insertion of foreign genes in gram-negative bacteria.* Journal of Bacteriology, 1990. **172**(11): p. 6557-6567.

6. Hanahan, D., *Studies on transformation of Escherichia coli with plasmids.* Journal of molecular biology, 1983. **166**(4): p. 557-580.

7. Zhang, Y., U. Werling, and W. Edelmann, *SLiCE: a novel bacterial cell extract-based DNA cloning method.* Nucleic acids research, 2012. **40**(8): p. e55-e55.

8. Li, M.Z. and S.J. Elledge, *SLIC: a method for sequence-and ligation-independent cloning*, in *Gene synthesis*. 2012, Springer. p. 51-59.

9. Larkin, M.A., et al., *Clustal W and Clustal X version 2.0.* bioinformatics, 2007. **23**(21): p. 2947-2948.

10. Stecher, G., K. Tamura, and S. Kumar, *Molecular evolutionary genetics analysis (MEGA) for macOS.* Molecular Biology and Evolution, 2020. **37**(4): p. 1237-1239.
